# Supplementary material for: The Genome Analysis of the Human Lung-Associated Streptomyces sp. TR1341 Revealed the Presence of Beneficial Genes for Opportunistic Colonization of Human Tissues
Source: Microorganisms. 2021 Jul 21;9(8):1547. doi: 10.3390/microorganisms9081547 (PMC8401907; doi:10.3390/microorganisms9081547)
Supplement: Supplementary file 1 [file microorganisms-09-01547-s001.zip › FigureS1.pdf]

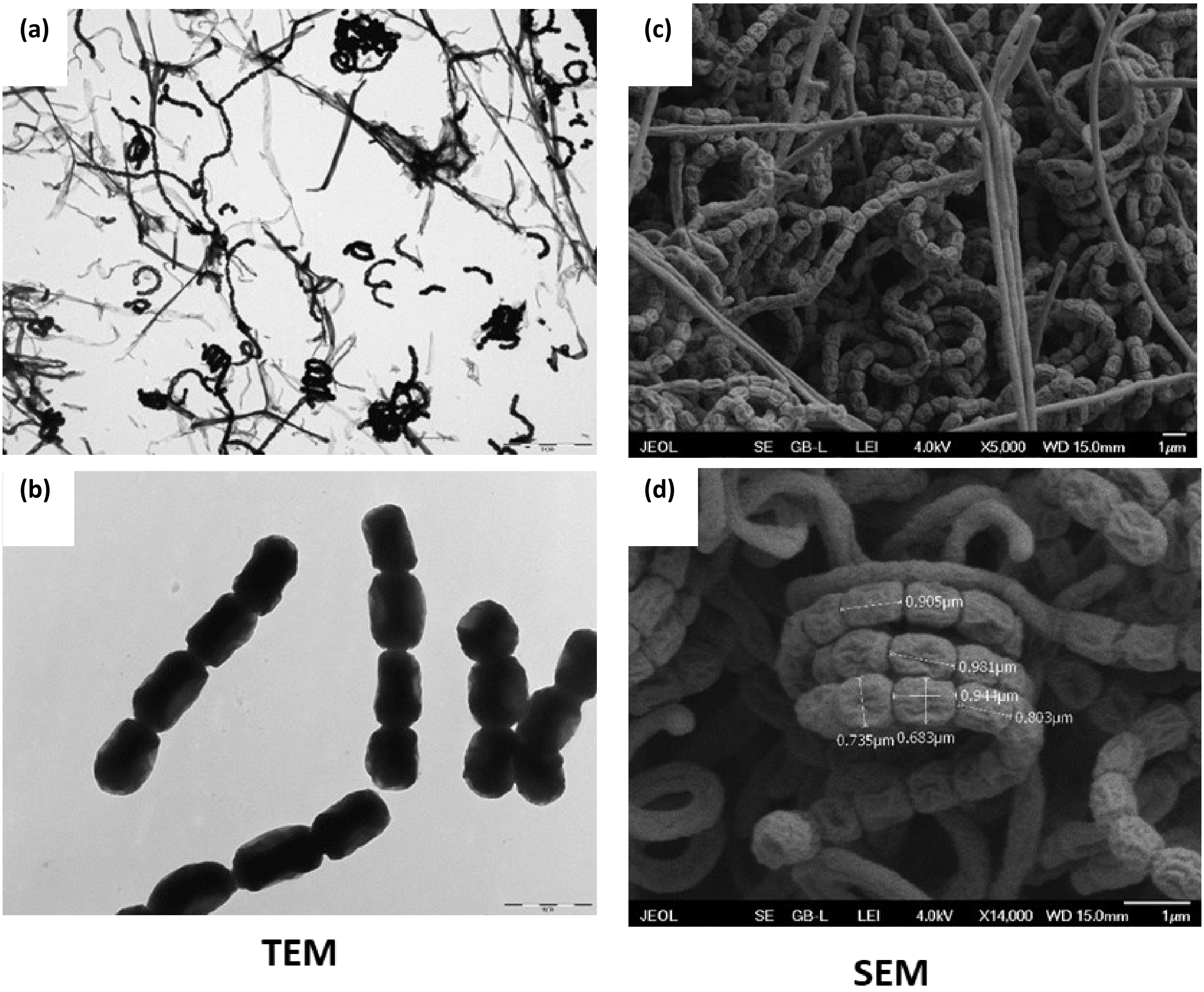

Figure S1. Electron microscopy images of *Streptomyces* sp. TR1341. (**a**,**b**) Transmission electron microscpy (TEM); (**c**,**d**) Scanning electron microscopy (SEM).
