# Supplementary material for: The Genome Analysis of the Human Lung-Associated Streptomyces sp. TR1341 Revealed the Presence of Beneficial Genes for Opportunistic Colonization of Human Tissues
Source: Microorganisms. 2021 Jul 21;9(8):1547. doi: 10.3390/microorganisms9081547 (PMC8401907; doi:10.3390/microorganisms9081547)
Supplement: Supplementary file 1 [file microorganisms-09-01547-s001.zip › FigureS2.pdf]

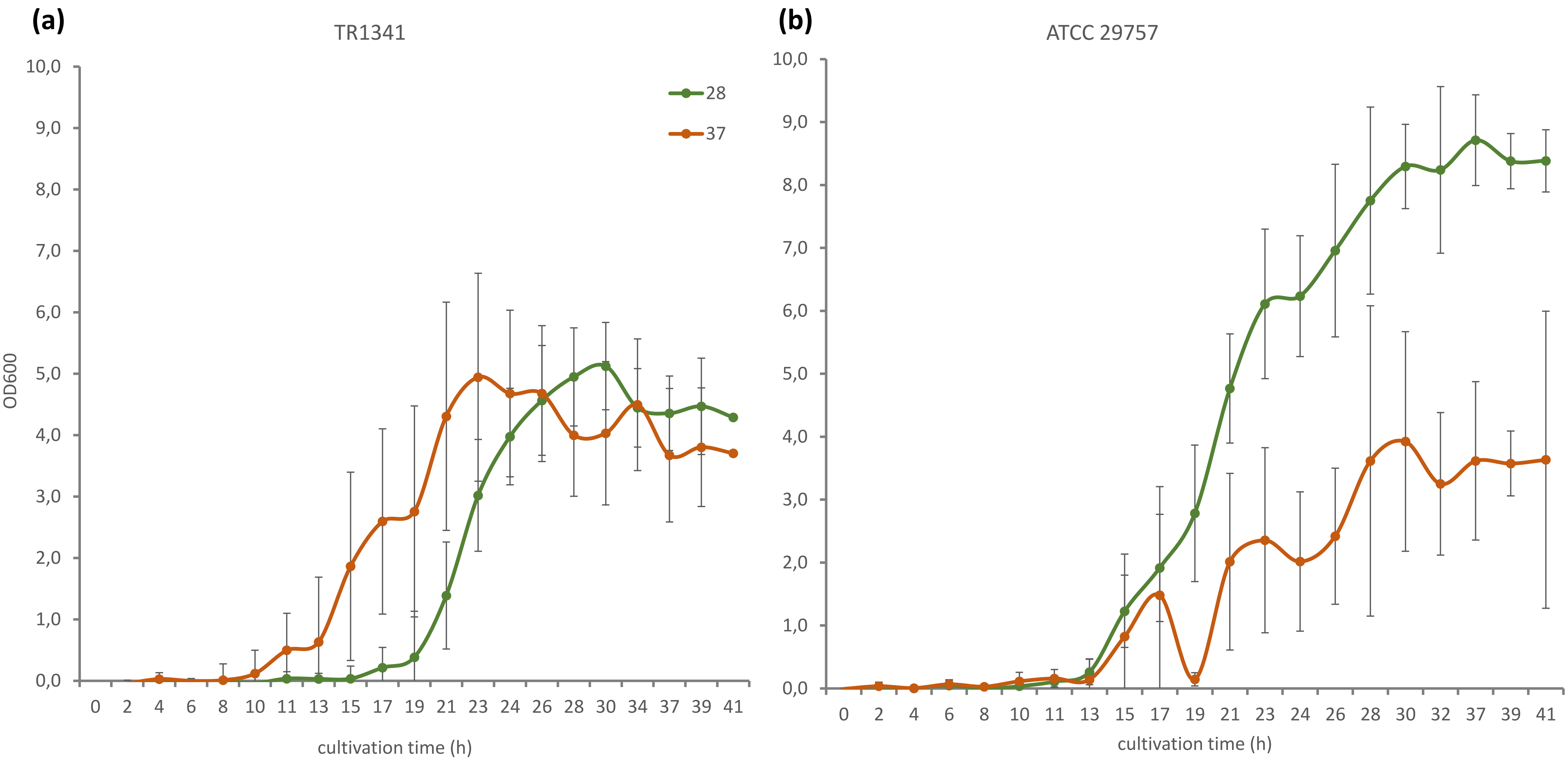

Figure S2. Growth curves of (**a**) *Streptomyces* sp. TR1341 and (**b**) *S. nodosus* ssp. *asukaensis* ATCC 29757 grown in LB media at 28°C (green) and 37°C (red). Average values of 3 biological replicates and standard deviations are reported.
