# Supplementary material for: The Genome Analysis of the Human Lung-Associated Streptomyces sp. TR1341 Revealed the Presence of Beneficial Genes for Opportunistic Colonization of Human Tissues
Source: Microorganisms. 2021 Jul 21;9(8):1547. doi: 10.3390/microorganisms9081547 (PMC8401907; doi:10.3390/microorganisms9081547)
Supplement: Supplementary file 1 [file microorganisms-09-01547-s001.zip › FigureS3.pdf]

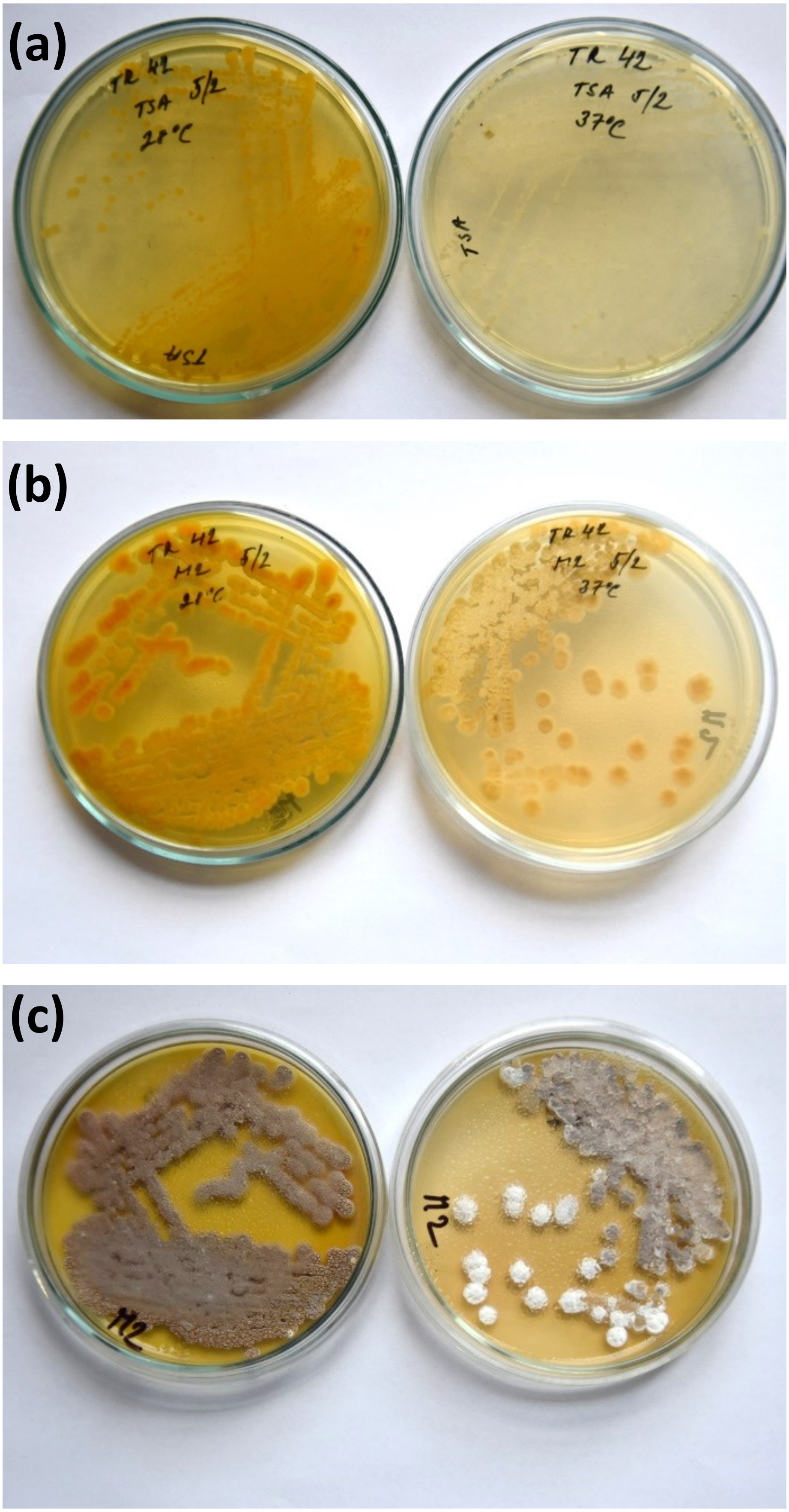

Figure S3. The effect of temperature on the growth characteristics of *Streptomyces* sp. TR1341. (**a**) TR1341 grown on TSA at 28°C (right) and 37°C (left); (**b**) and (**c**) back and front of the plates with TR1341 grown on M2 at 28°C (right) and 37°C (left), respectively.
