# Supplementary material for: The Genome Analysis of the Human Lung-Associated Streptomyces sp. TR1341 Revealed the Presence of Beneficial Genes for Opportunistic Colonization of Human Tissues
Source: Microorganisms. 2021 Jul 21;9(8):1547. doi: 10.3390/microorganisms9081547 (PMC8401907; doi:10.3390/microorganisms9081547)
Supplement: Supplementary file 1 [file microorganisms-09-01547-s001.zip › FigureS6.pdf]

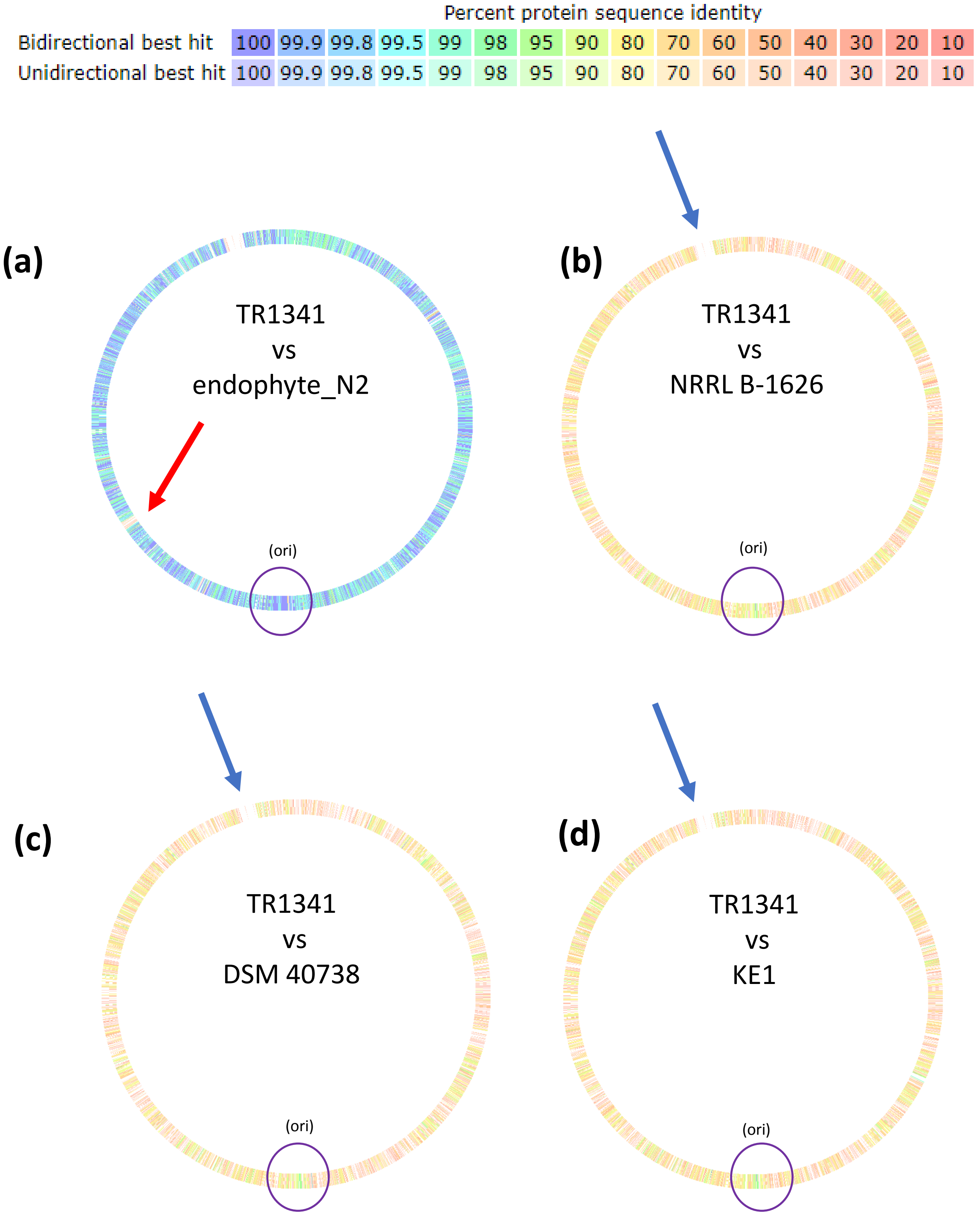

Figure S6.: Comparison of *Streptomyces* sp. TR1341 with *Streptomyces* sp. endophyte_N2 and human-associated streptomycetes at amino acid level. Bidirectional best hit calculated in RAST. TR1341 vs (**a**) *Streptomyces* sp. endophyte_N2, (**b**) *Streptomyces brasiliensis* NRRL B-1626, (**c**) *Streptomyces somaliensis* DSM 40738, (**d**) *Streptomyces* sp. KE1. Red arrow indicates a region (red region) of the genome that has low identity between TR1341 and *Streptomyces* sp. endophyte_N2. Blue arrows indicate a region (blue region) that seems to be unique in TR1341 when compared with the human-associated streptomycetes.
