# Supplementary material for: The Genome Analysis of the Human Lung-Associated Streptomyces sp. TR1341 Revealed the Presence of Beneficial Genes for Opportunistic Colonization of Human Tissues
Source: Microorganisms. 2021 Jul 21;9(8):1547. doi: 10.3390/microorganisms9081547 (PMC8401907; doi:10.3390/microorganisms9081547)
Supplement: Supplementary file 1 [file microorganisms-09-01547-s001.zip › FigureS7.pdf]

(a) TR1341 vs endophyte_N2: Red Region

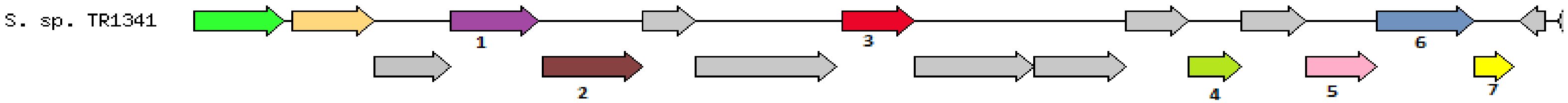

1) Vanillate O-demethylase oxygenase subunit (EC 1.14.13.82),2) S-adenosylmethionine synthetase (EC 2.5.1.6), 3) O-methyltransferase,4) LysR-family transcriptional regulatory protein, 5) SAM-dependent methyltransferase, 6) Chorismate synthase (EC 4.2.3.5),7) Shikimate kinase I (EC 2.7.1.71) and hypothetical proteins.

(b) TR1341 vs human associated *Streptomyces:* Blue Region

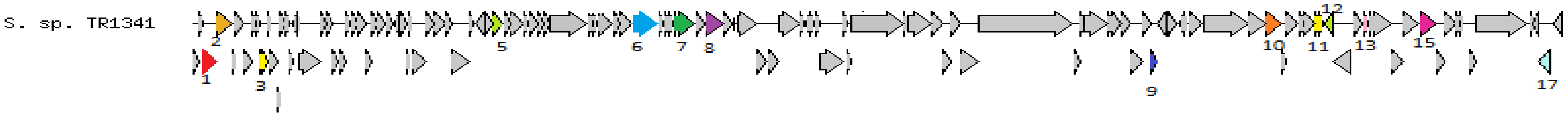

1)phage-related protein, 2) Recombinational DNA repair protein RecT, 3)Sporulation regulatory protein WhiD(prophage associated), 4)Putative DNA helicase, 5) Protein serine/threonine phosphatase PrpC regulation of stationary, 6) C-5 cytosine-specific DNA methylase family protein, phase, 7) MG(2+) CHELATASE FAMILY PROTEIN / ComM-related protein, 8)Chromosome (plasmid) partitioning protein ParB, 9) Phage protein (ACLAME 479), 10)Sulfur carrier protein adenylyltransferase ThiF, 11) Phage protein, 12) acetyltransferase, 13) Putative C1 regulatory protein, 14) Uncharacterized RsbU-domain-containing protein SCO5040, 15) Radical SAM domain protein, 16) hypothetical protein possible MFS-type secretion effector / Thymidylate kinase (EC 2.7.4.9), 17) Rhs-family protein

Figure S7. Dissimilar genomic regions of *Streptomyces* sp. TR1341 with *Streptomyces* sp. endophyte_N2 and human associated *Streptomyces*. (**a**) TR1341 vs *Streptomyces* sp. endophyte_N2; (**b**) TR1341 vs human associated *Streptomyces.* The annotation of the colored genes is listed below each figure. Grey blocks represent hypothetical proteins.
