# Supplementary material for: The Genome Analysis of the Human Lung-Associated Streptomyces sp. TR1341 Revealed the Presence of Beneficial Genes for Opportunistic Colonization of Human Tissues
Source: Microorganisms. 2021 Jul 21;9(8):1547. doi: 10.3390/microorganisms9081547 (PMC8401907; doi:10.3390/microorganisms9081547)
Supplement: Supplementary file 1 [file microorganisms-09-01547-s001.zip › FigureS8.pdf]

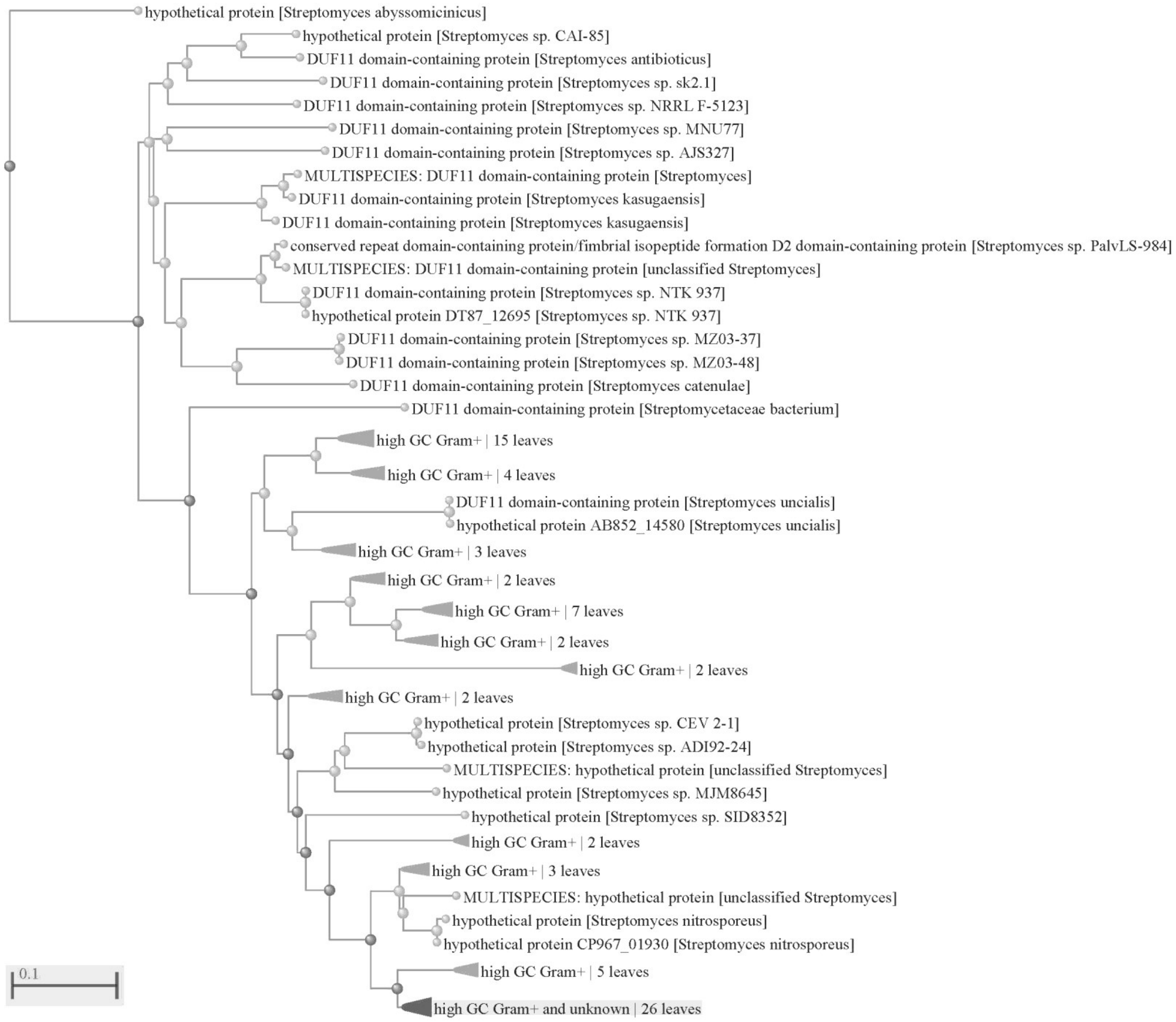

Figure S8. Distribution and similarity of genes with DUFF11 conserved domain among *Streptomyces* genomes available in the NCBI database.
